# PERSON-CENTERED CARE PLANNING FOR PERSONS WITH MCC: LESSONS FROM LEARNING COLLABORATIVE DISCUSSIONS

**DOI:** 10.1093/geroni/igae098.1862

**Published:** 2024-12-31

**Authors:** Lyndsey Miller, Emilia DeMarchis, LeAnn Michaels, Christie Jaskson, David Dorr

**Affiliations:** Oregon Health & Science University, Portland, Oregon, United States; University of California San Francisco, San Francisco, California, United States; Oregon Health & Science University, Portland, Oregon, United States; Oregon Health & Science University, Portland, Oregon, United States; Oregon Health & Science University, Portland, Oregon, United States

## Abstract

The Learning Collaborative invites person-centered care planning (PCCP) innovators, implementation experts, and front-line providers to share and discuss successes and challenges they have experienced in PCCP with the goal of generating a framework for increasing the uptake of and scaling PCCP as an integral component of routine practice. The Learning Collaborative reviews promising models of PCCP and describes feasible solutions to common implementation barriers. Participants engage in deliberative dialogue based on the collective experiences of the group to generate new knowledge and identify best practices and strategies to deliver high-quality PCCP for persons with MCCs. This task involves a series of 5 Learning Collaborative sessions to review and discuss case studies of PCCP. The Learning Collaborative includes 25-30 participants affiliated with partner Practice-Based Research Networks (PBRNs), recommended from the Technical Expert Panel, and identified by AHRQ. Learning Collaborative participants include individuals with front-line experience implementing PCCP; health system or practice clinicians preparing to implement PCCP; and implementation science researchers with experience implementing PCCP. Gathering all these partners will permit shared learning to accelerate progress, iterative, collective discussions to develop a framework for successful implementation of PCCP for people living with or at risk for MCC. The synthesis of learnings and reporting from the Learning Collaborative will provide key information for change agents, such as policymakers, clinical teams, and health care system leaders wishing to implement and research PCCP.

